# The Impact of COVID-19 Outbreak on Emotional and Cognitive Vulnerability in Iranian Women With Breast Cancer

**DOI:** 10.3389/fpsyg.2021.663310

**Published:** 2021-05-31

**Authors:** Mohammad H. Choobin, Vida Mirabolfathi, Bethany Chapman, Ali Reza Moradi, Elizabeth A. Grunfeld, Nazanin Derakshan

**Affiliations:** ^1^Department of Clinical Psychology, Kharazmi University, Tehran, Iran; ^2^Department of Cognitive Psychology, Institute for Cognitive Science Studies (ICSS), Tehran, Iran; ^3^Centre for Building Resilience in Breast Cancer; Department of Psychological Sciences, Birkbeck, University of London, London, United Kingdom

**Keywords:** breast cancer, COVID-19, emotional vulnerability, cognitive function, psychological health

## Abstract

The psychological cost on emotional well-being due to the collateral damage brought about by COVID-19 in accessing oncological services for breast cancer diagnosis and treatment has been documented by recent studies in the United Kingdom. The current study set out to examine the effect of delays to scheduled oncology services on emotional and cognitive vulnerability in women with a breast cancer diagnosis in Iran, one of the very first countries to be heavily impacted by COVID-19. One hundred thirty-nine women with a diagnosis of primary breast cancer answered a series of online questionnaires to assess the current state of rumination, worry, and cognitive vulnerability as well as the emotional impact of COVID-19 on their mental health. Results indicated that delays in accessing oncology services significantly increased COVID related emotional vulnerability. Regression analyses revealed that after controlling for the effects of sociodemographic and clinical variables, women’s COVID related emotional vulnerability explained higher levels of ruminative response and chronic worry as well as poorer cognitive function. This study is the first in Iran to demonstrate that the effects of COVID-19 on emotional health amongst women affected by breast cancer can exaggerate anxiety and depressive related symptoms increasing risks for clinical levels of these disorders. Our findings call for an urgent need to address these risks using targeted interventions exercising resilience.

## Introduction

Iran was one of the first countries to be significantly affected by the coronavirus disease (SARS-CoV-2; COVID-19) outbreak shortly after China. The rapid spread of the virus throughout the country caused profound psychological fear (Pakpour and Griffiths, 2020), anxiety (Moghanibashi-Mansourieh, 2020) and resulted in many restrictions to the regular medical health care services available including, cancer clinics.

Breast cancer is the most common cancer in Iran with the mean age of 49.84 (Nafissi et al., 2018) and an incidence rate of 28.3 per 100,000 in women (Sharifian et al., 2015). Throughout diagnosis, treatment and well into survivorship, women require continuous access to multiple healthcare services (Dietz et al., 2020).

There are no published studies on the impact of COVID-19 on rates of recurrence and survival in breast cancer. However, a recent report has revealed a series of temporary modifications have been made to the approved breast cancer care guidelines in Iran to reserve medical resources for individuals with COVID-19 (Shahi et al., 2020). Studies now show that the United Kingdom has witnessed a 15% decline in breast cancer referrals during 2020 compared to a similar period in 2019 (Gathani et al., 2021), with estimations for significant reductions in survival rates and increased mortality as a result of the collateral damage brought about by COVID-19 on cancer treatment (see Maringe et al., 2020). These changes in circumstances created by the COVID-19 outbreak has imposed a great deal of distress and trauma on healthcare providers who have been forced to make unprecedented clinical decisions and handle reorganization, including treatment allocation and modification (Romeo et al., 2020).

In a recent meta-analysis that investigated the survival rate of women with primary breast cancer, both age at diagnosis and stage of breast cancer independently affected survival rate (Maajani et al., 2019). Studies show that delays in early diagnosis and disruption to active treatment can severely reduce survival rates (Smith et al., 2013). In younger women aged <45 years, breast cancer is more aggressive with a less favorable prognosis, and worse mortality rate than in older women (Radecka and Litwiniuk, 2016). It is also well documented that younger women diagnosed with breast cancer not only suffer from long term physical problems such as fatigue, insomnia, joint pain, and hot flushes but also tolerate radical early menopause and loss of fertility caused by chemotherapy and endocrine therapies (Rosenberg and Partridge, 2013; Anastasiadi et al., 2017) such as Tamoxifen. Given, the high prevalence of younger women (<45 years) diagnosed with aggressive breast cancer in Iran the disruption to available healthcare service caused by the pandemic could have a profound effect on breast cancer mortality over the next few years.

A large and growing body of literature substantiates that cancer-related cognitive impairments (CRCI) (see Ahles and Root, 2018, for a review) can exist for many years post active treatment substantially affecting the quality of life. Self-reported cognitive function plays a critical role in determining emotional well-being in women with breast cancer (Chapman et al., 2019). Cognitive mechanisms such as rumination and worry increase an individual’s vulnerability to developing distinct and overlapping forms of emotional disorders such as anxiety and depression (see Koster et al., 2017, for a review). In a recent meta-analysis by Wang et al. (2020), which included 282,203 breast cancer patients, it was found that depression and anxiety independently predicted all-cause mortality and cancer recurrence, respectively. It is imperative therefore that the risk factors for these disorders are understood. Rumination is conceptualized as repetitive negative affective thoughts focused on current or past events (Nolen-Hoeksema, 2000), and worry is identified by involuntary thoughts about possible future threats (Borkovec et al., 1983). Whilst intrusive rumination has a critical role in the initiation and maintenance of Post Traumatic Stress Disorder (PTSD) symptoms in women living with a diagnosis of breast cancer (Ogińska-Bulik and Michalska, 2020), worry has been shown to be more strongly associated with depression and anxiety in cancer survivors (Brown et al., 2018). Ruminative thinking can jeopardize well-being in patients diagnosed with primary breast cancer (Liu et al., 2020). Further, a recent study by Renna et al. (2020) indicated that both rumination and worry increase cancer-related distress and indirectly result in more self-reported physical complaints in women with a history of breast cancer. It is, therefore, not surprising that disruptions in medical services due to the COVID-19 outbreak could not only result in more severe physical conditions in breast cancer patients but also lead to more acute emotional distress and worse cognitive impairment (Swainston et al., 2020). It is also a critical risk for developing PTSD symptoms (Romeo et al., 2020).

The current study aimed to firstly investigate the impact of delays in accessing medical care on levels of COVID related emotional vulnerability (COVID-EMV). Secondly, by considering the critical role that age plays in treatment outcomes in cancer patients (Fernandes-Taylor et al., 2015), we investigated the impact of age on rumination, worry, cognitive function, and COVID-EMV. We then examined the relationship between COVID-19 related emotional vulnerability (COVID-EMV), rumination, worry, and cognitive function whilst allowing for clinical and sociodemographic variables (education, grade, age at diagnosis, time since treatment finished, and health co-morbidity).

Firstly, we predicted that women who experienced delays in accessing medical care would report higher COVID-EMV compared to women who experienced no disruption. Secondly, we predicted that younger women (≤45 years old a cut-off recommended by Breast cancer in younger women, 2018) would experience more severe rumination, pathological worry, cognitive dysfunction and COVID-EMV compared to older women (>45 years old). We expected that after allowing for a range of clinical and sociodemographic variables COVID-EMV would significantly predict higher levels of trait vulnerability to emotional disorders as documented through higher rumination, worry and poorer cognitive function.

## Materials and Methods

### Design

A cross-sectional approach was used to investigate the impact of the COVID-19 outbreak on the emotional well-being and cognitive function of women living with a breast cancer diagnosis in Iran. Participants were asked to respond to a series of online questionnaires assessing their demographic characteristics such as type of breast cancer, medical history, level of rumination, pathological worry and cognitive function.

### Participants

A total of 139 women with a diagnosis of primary breast cancer (*Mean Age* = *45; SD* = *9.60, Mean Age at Diagnosis* = *42, SD* = *9.38)* were voluntarily recruited via multiple sources: online advertisement placed on social media platforms including, Instagram pages related to breast cancer and COVID-19 (such as @Coronavirus_breast_cancer owned by researchers) and the cancer research center of Imam Khomeini Hospital in Tehran. The primary inclusion criteria for participants was a diagnosis of primary breast cancer. Participants were recruited between May 9th 2020 and May 29th 2020, during Iran’s first pandemic peak.

### Materials

#### Demographic and Clinical Questionnaire (DQ)

To collect participant’s information regarding their breast cancer history and sociodemographic characteristics we designed a 27 item self-report questionnaire (see Table 1).

**TABLE 1:** Participant demographics, clinical characteristics, and psychiatric history.

Demographic variable	*N* = 139 (%)
Current age	Mean = 45.86 years (Min = 17, Max = 75)
Age at diagnosis	Mean = 42.43 years (Min = 16, Max = 74)
Time on treatment	Mean = 12.76 months (Min = 1, Max = 146)
Time since treatment finished	Mean = 27.32 months (Min = 0, Max = 199)
**Age category**	
Less than or equal to 45	71 (51.1%)
More than 45	68 (48.9%)
**Education**	
8 Years or less	17 (12.2%)
Diploma or college	38 (27.3%)
Undergraduate	66 (47.5%)
M.Sc or Ph.D.	18 (12.9%)
**Civil status**	
Single	13 (9.4%)
Married	117 (84.2%)
Divorced	9 (6.5%)
**Employment status**	
Private or public sector employee	36 (25.9%)
Self-employment	16 (11.5%)
Studying	3 (2.2%)
Unemployed	68 (48.9%)
Retired	16 (11.5%)
Active treatment	26 (18.7%)
**Grade**	
Grade I	30 (21.6%)
Grade II	54 (38.8%)
Grade III	55 (39.6%)
Lymph node involvement	63 (45.3%)
**Type of treatment received**	
Chemotherapy	108 (77.7%)
Radiotherapy	97 (69.8%)
**Surgery**	
Mastectomy	84 (60.4%)
Lumpectomy	34 (24.5%)
Mastectomy and lumpectomy	3 (2.2%)
Endocrine therapy	74 (53.2%)
Delays in accessing medical care	38 (27.3%)
Delays in surgery	4 (2.9%)
Delays in treatment	34 (24.5%)

#### Functional Assessment of Cancer Therapy-Cognitive Scale (FACT-Cog)

The third version of the FACT-Cog (Wagner et al., 2009) is a 37-item questionnaire, using a 5-point Likert-scale, with a total score ranging from 0 to 148. It consists of four subscales: perceived cognitive impairments (PCI, 20 items), comments from others (4 items), perceived cognitive ability (PCA, 9 items), and impact on quality of life (QoL, 4 items). This questionnaire is designed to measure cognitive complaints and abilities in the past 7 days. Higher scores in all subscales indicate better cognitive function and quality of life. This highly reliable questionnaire has been widely used in previous breast cancer studies (Von Ah and Tallman, 2015; Janelsins et al., 2017; Vardy et al., 2019). A Farsi version of this questionnaire with excellent internal consistency was used in this study (α = 0.97).

#### Rumination Response Scale (RRS)

The rumination scale includes 22 items that assess the depressive rumination experienced over the last 7 days. Each item is rated on a Likert scale ranging from 1 (almost never) to 4 (almost always), with total scores ranging from 22 to 88. Higher scores indicate more rumination. The Internal consistency for the Farsi version is α = 0.88 (Bagherinezhad et al., 2010). This reliable questionnaire has been used in many cancer research studies in Iran (Heidarian et al., 2016). The internal consistency in the present study was excellent (α = 0.96).

#### Penn State Worry Questionnaire (PSWQ)

The PSWQ is a 16-item inventory with a 5-point Likert scale (1 = Not at all typical of me, 5 = very typical of me), higher scores represent a greater level of pathological worry (PSWQ total score ranges from 16 to 80). The PSWQ is a widely used measure in breast cancer research (Ghanavati et al., 2018; Swainston and Derakshan, 2018; Chapman et al., 2020). In this study, a Farsi version with a good internal consistency was used (α = 0.88) (Dehshiri et al., 2010). Internal consistency in the current study was 0.91.

#### Modified Self-Report-Generated Charlson Comorbidity (CCI)

The modified version of the Charlson Comorbidity Index with nine comorbidity items was used to measure pre-existing health conditions. All participants were asked to respond to this questionnaire based on their knowledge of their current medical conditions. These nine items are given independent weighted values (i.e., HIV illness or AIDS = 6, Diabetes = 2). The CCI total score ranges from 0 to 19 by the summation of these values. A higher score specifies more chronic comorbidity. The CCI has been used in previous research to measure comorbidity in women diagnosed with breast cancer (Ording et al., 2013; Fu et al., 2015; Soleymanian and Ghaziani, 2018). In this study, the summed score of CCI was used.

#### COVID-19 Impact Questions

Twenty-three individual items were translated from Swainston et al. (2020) and Chapman et al. (2020) to assess the impact of the COVID-19 outbreak in women living with breast cancer in Iran. Specifically, questions examined the impact of COVID-19 on current emotional (psychological) vulnerability as well as the history of the COVID-19 symptoms (i.e., high temperature/fever, continuous cough).

To measure the emotional impact of COVID-19, a composite score was formed by combining the five psychological related items (i.e., the outbreak led me to feel more: (1) anxious, (2) upset, (3) fearful than usual or has the outbreak made you feel less: (4) in control or (5) less confident than usual). This composite COVID emotional vulnerability (COVID-EMV) score showed an excellent internal consistency (α = 0.90) (see Supplementary Material for more detailed factor analysis and COVID-EMV correlations).

In the COVID-19 symptoms section, personal experience of COVID-19 symptoms was assessed by checking the COVID-19 symptoms confirmed by World Health Organsiation (2020) (high temperature/fever, continuous cough, shortness of breath, chest pain or pressure, sore throat, sneezing or runny nose, loss of smell and/or taste, bluish lips or face or new confusion or inability to arouse). In addition, isolation status and any change in cancer treatment plan due to the outbreak were individually asked.

### Procedure

Before completing the online questionnaires, participants were asked to provide a telephone number to receive a phone call from the researchers to become familiar with the structure of the study and its overall aim. Once the participant agreed to participate in the research, they received the research URL code via an email to access the battery of self-report questionnaires. Following completing the online consent form, they were directed to the DQ, CCI, FACT-Cog, RRS, PSWQ and finally the COVID-19 Impact Questions. At the end of the session, participants were thanked for their time and effort, with 500,000 Rials transferred to their bank account. Before commencing the study, ethical approval was obtained from the Research Ethics Committee of the Kharazmi University (Ethic code: IR.KHU.REC.1399.002).

### Statistical Analysis

All analyses were carried out using R, version 4.0.3. Descriptive statistics were calculated for sociodemographic characteristics and history of breast cancer (see Table 1). Independent *t*-tests were conducted to explore the impact of delays in accessing medical care (i.e., delay vs. no delay) on COVID-EMV as well as the effects of age on rumination, worry, cognitive function and COVID-EMV (see Table 2). Cohen’s *d* effect sizes were calculated.

**TABLE 2:** *t*-test results in comparing the age groups^*a*^.

	Age ≤ 45 (*n* = 71)	Age > 45 (*n* = 68)		
	Mean (*SD*)	Mean (*SD*)	*t*	*P*
Rumination	49.49 (16.71)	44.25 (14.01)	2.00	0.05
Pathological worry	50.68 (14.38)	51.12 (13.85)	–0.18	0.85
Cognitive function	106.25 (26.43)	110.16 (28.12)	–0.84	0.40
COVID-EMV	13.37 (5.55)	10.97 (5.08)	2.65	0.01

**^*a*^Rumination: higher score = more rumination; Pathological worry: higher score = greater worry; Cognitive function: higher score = better cognitive function; COVID-EMV: higher score = more emotional vulnerability.**

Hierarchical regression analyses were run on the total sample size (*N* = 139) to assess the relationship between COVID-EMV and rumination (see Table 3), pathological worry (see Table 4), and cognitive ability (see Table 5). On the first step, five factors were added including, education, grade, age at diagnosis, time since treatment finished, and CCI. COVID-EMV was then entered on the second step.

**TABLE 3:** Hierarchical regression analyses for the predictors of rumination.

	*b*	*SE B*	*β*	*t*	*p*
**Step 1**					
(Intercept)	62.53 (43.46, 81.60)	9.64		6.49	0.00
Education	−1.78 (−.94, 1.39)	1.60	–0.10	–1.11	0.27
Grade	2.19 (−1.23, 5.60)	1.73	0.11	1.27	0.21
Age at diagnosis	−0.38 (−0.68, −0.08)	0.15	–0.23	–2.51	0.01
Time since treatment finished	−0.04 (−0.11, 0.02)	0.03	–0.11	–1.28	0.20
Charlson Co-morbidity Index	2.55 (0.35, 4.75)	1.11	0.20	2.30	0.02
Multiple-R^2^		0.09			
Adjusted-R^2^		0.06			
F-change		2.75			0.02
**Step 2**					
(Intercept)	35.39 (18.56, 52.22)	8.51		4.16	0.00
Education	−1.26 (−3.84, 1.32)	1.30	–0.07	–0.96	0.34
Grade	0.75 (−2.06, 3.55)	1.42	0.04	0.53	0.60
Age at diagnosis	−0.20 (−0.45, 0.04)	0.12	–0.12	–1.63	0.11
Time since treatment finished	0.00 (−0.05, 0.06)	0.03	0.01	0.11	0.91
Charlson Co-morbidity Index	2.29 (0.50, 4.08)	0.91	0.18	2.53	0.01
COVID-EMV	1.67 (1.27, 2.06)	0.20	0.58	8.28	0.00
Multiple-R^2^		0.40			
Adjusted-R^2^		0.38			
F-change		14.89			0.00

**95% confidence intervals.**

**TABLE 4:** Hierarchical regression analyses for the predictors of pathological worry.

	*b*	*SE B*	*β*	*t*	*p*
**Step 1**					
(Intercept)	48.32 (30.46, 66.18)	9.03		5.35	0.00
Education	−1.53 (−4.49, 1.44)	1.50	–0.09	–1.02	0.31
Grade	1.64 (−1.56, 4.84)	1.62	0.10	1.02	0.31
Age at diagnosis	0.07 (−0.21, 0.35)	0.14	0.04	0.47	0.64
Time since treatment finished	0.00 (−0.06, 0.07)	0.03	0.00	0.05	0.96
Charlson Co-morbidity Index	0.17 (−1.89, 2.23)	1.04	0.01	0.17	0.87
Multiple-R^2^		0.02			
Adjusted-R^2^		–0.02			
F-change		0.58			0.71
**Step 2**					
(Intercept)	26.71 (9.86, 43.57)	8.52		3.14	0.01
Education	−1.11 (−3.70, 1.47)	1.31	–0.07	–0.85	0.40
Grade	0.50 (−2.31, 3.31)	1.42	0.03	0.35	0.73
Age at diagnosis	0.21 (−.04, 0.45)	0.13	0.14	1.65	0.10
Time since treatment finished	0.04 (−0.02, 0.10)	0.03	0.11	1.37	0.17
Charlson Co-morbidity Index	−0.04 (−1.83, 1.76)	0.91	–0.01	–0.04	0.97
COVID-EMV	1.33 (0.93, 1.72)	0.20	0.51	6.58	0.00
Multiple-R^2^		0.26			
Adjusted-R^2^		0.23			
F-change		7.86			0.00

**95% confidence intervals.**

**TABLE 5:** Hierarchical regression analyses for the predictors of perceived cognitive function.

	*b*	*SE B*	*β*	*t*	*p*
**Step 1**					
(Intercept)	91.88 (58.67, 125.08)	16.79		5.47	0.00
Education	4.31 (−1.20, 9.82)	2.79	0.14	1.55	0.12
Grade	−3.57 (−9.52, 2.38)	3.01	–0.10	–1.19	0.24
Age at diagnosis	0.34 (−0.18, 0.86)	0.26	0.12	1.29	0.20
Time since treatment finished	0.06 (−0.06, 0.18)	0.06	0.09	1.01	0.31
Charlson Co-morbidity Index	−5.40 (−9.22, −1.57)	1.93	–0.24	–2.79	0.01
Multiple-R^2^		0.10			
Adjusted-R^2^		0.06			
F-change		2.86			0.02
**Step 2**					
(Intercept)	123.05 (89.73, 156.38)	16.85		7.30	0.00
Education	3.72 (−1.39, 8.82)	2.58	0.12	1.44	0.15
Grade	−1.92 (−7.48, 3.63)	2.81	–0.05	–0.69	0.49
Age at diagnosis	0.14 (−0.35, 0.63)	0.25	0.05	0.56	0.58
Time since treatment finished	0.01 (−0.11, 0.12)	0.06	0.01	0.12	0.91
Charlson Co-morbidity Index	−5.10 (−8.64, −1.55)	1.79	–0.23	–2.84	0.01
COVID-EMV	−1.91 (−2.70, −1.13)	0.40	–0.38	–4.80	0.00
Multiple-R^2^		0.23			
Adjusted-R^2^		0.20			
F-change		6.62			0.00

**95% confidence intervals.**

Preliminary analyses were conducted to ensure no violation of the assumptions of normality, linearity, and homoscedasticity. Using analysis of standardized residual, no outliers were found (Rumination: std Residual Min = −2.64, std Residual Max = 2.84, std deviation = 0.98; Worry: std Residual Min = −2.11, std Residual Max = 2.73, std deviation = 0.98; Perceived cognitive function: std Residual Min = −3.16, std Residual Max = 1.98, std deviation = 0.98). *Post hoc* analyses were conducted using Cohen’s *f*^2^ (Ben-Shachar et al., 2020) and a significance of 0.05.

There was no missing data for any of the questionnaires used in the current study.

## Results

Data was collected from 139 women with a primary breast cancer diagnosis (*mean age* = 45.86, *SD* = 9.60; *mean age at diagnosis* = 42.43, *SD* = 9.38). A total of 38 women (27.3%) had been impacted by disruption to their scheduled medical services [delay in surgery (2.9%) and delay in treatment (24.5%)]. Only 32 women (23%) reported that they had shown COVID-19 related symptoms (7.9% of symptoms reported were fever and/or cough). 109 participants (78.4%) revealed that they had decided to self-isolate themselves to reduce the risk of contracting the virus. None of the participants reported that they had received a clinical diagnosis of COVID-19.

### Effects of Delay

Of the 139 women who completed the questionnaire, 38 (27.3%) experienced delays in accessing medical care (4 experienced delays to surgery and 34 experienced delays to treatment). This group reported significantly more COVID-EMV (*M* = 13.66, *SD* = 5.99) compared to those who reported no delays (*M* = 11.64, *SD* = 5.14; *t* (137) = 1.97, *p* = 0.05, *d* = 0.37]. Women who had experienced delays reported significantly more pathological worry (*M* = 54.79, *SD* = 13.39) compared to those who experienced no delays (*M* = 49.43, *SD* = 14.11; *t* (137) = 2.02, *p* = 0.04, *d* = 0.39]. These effects were not visible for rumination scores [*t* (137) = 1.28, *p* = 0.20, *d* = 0.24] between women in the delayed group (*M* = 49.68, *SD* = 13.44) and the no-delay group (*M* = 45.89, *SD* = 16.30). There were no group differences on perceived cognitive ability (delayed group *M* = 103.16, *SD* = 27.39; no delay group: *M* = 110.05, *SD* = 27.08; *t* (137) = −1.33, *p* = 0.18, *d* = 0.2).

### Effects of Age

As shown in Table 2, younger women (current age ≤ 45 years) reported significantly more rumination (*M* = 49.49, *SD* = 16.71) than older women (*M* = 44.25, *SD* = 14.01; *t* (137) = 2.00, *p* = 0.05, *d* = 0.34). Younger women also reported significantly more COVID-EMV (*M* = 13.37, *SD* = 5.55) compared with older women (*M* = 10.97, *SD* = 5.08; *t* (137) = 2.65, *p* = 0.01, *d* = 0.45). There were no significant differences in pathological worry (*t* (137) = −0.18, *p* = 0.85, *d* = 0.03) for younger women (*M* = 50.68, *SD* = 14.38) and older women (*M* = 51.12, *SD* = 13.85), and also in cognitive function (*t* (137) = −0.84, *p* = 0.40, *d* = 0.14) between younger women (*M* = 106.25, *SD* = 26.43) and older women (*M* = 110.16, *SD* = 28.12).

### Impact of Covid-19 Related Distress on Emotional Vulnerability

#### Rumination

On Step 1 of the hierarchical regression analysis, education, grade, age at diagnosis, time since treatment finished, and CCI accounted for 9.4% of the variance (see Table 3). When COVID-EMV was added on Step 2, it significantly predicted increased variance in rumination with an R^2^ (change) of 31% [*t* (132) = 8.28, *p* < 0.001], with higher COVID-EMV meeting higher rumination. CCI was also a significant predictor on the second step [*t* (132) = 2.53, *p* = 0.01]. Cohen’s *f*^2^ = 0.52 and achieved statistical power (1-ß err prob) was 0.99.

#### Pathological Worry

When predicting worry the demographic variables entered on Step 1 accounted for a small 2.2% variance in women’s pathological worry. The explained variance was increased to 26% when COVID-EMV was entered in step two (*t* (132) = 6.58, p < 0.001). Greater COVID-EMV met with a higher level of worry. COVID-EMV was the only significant predictor on the second step. Cohen’s *f*^2^ = 0.33 and achieved statistical power (1-ß err prob) was 0.99.

#### Cognitive Function

When predicting cognitive function our predictors on Step 1 included education, grade, age at diagnosis, time since treatment finished, and CCI, which collectively explained 9.7% of the variance in cognitive function. On the second step, COVID-EMV predicted significant variance in cognitive function with an R^2^ (change) of 13.3% [*t* (132) = −4.80, *p* < 0.001] such that lower cognitive function met a higher level of COVID-EMV. CCI was also a significant predictor on the second step [*t* (132) = −2.84, *p* = 0.01]. Cohen’s *f*^2^ = 0.17 and achieved statistical power (1-ß err prob) was 0.99.

Checks for violation of assumptions showed collinearity (Tolerance > 0.1, VIF < 10), independent error (Rumination: Durbin–Watson = 1.78; Pathological Worry: Durbin–Watson = 1.97; Perceived Cognitive Function: Durbin–Watson = 1.99) normality and homogeneity of variance and linearity were met for rumination, pathological worry and cognitive function.

## Discussion

The present study investigated the effect of the emotional distress caused by COVID-19 outbreak on levels of rumination and worry as well as cognitive function in women living with primary breast cancer in Iran. We found that those who experienced delays in accessing medical care had higher levels of COVID-19 related emotional distress and pathological worry. This finding is consistent with that of Swainston et al. (2020) who found that this disruption resulted in more general anxiety, depression and COVID-19 related emotional vulnerability in the United Kingdom’s breast cancer population. It suggests that factors associated with the COVID-19 pandemic (i.e., delays in surgery and treatment) have led to more excessive COVID-19 related emotional distress and worry in the breast cancer population.

Of focal importance we found that younger women (≤45 years old) reported more severe rumination (i.e., more repetitive negative thoughts) and COVID-EMV (i.e., more fear about cancer than usual feeling less control over their health) compared to older women (>45 years old). These findings could be partly driven by the fact that younger women are at a greater risk women are at a greater risk of having more aggressive tumor characteristics (i.e., Her2+), persistent fear of recurrence, metastasis and premature mortality (Anders et al., 2009; Assi et al., 2013; Champion et al., 2014; Lee et al., 2015) compared to older women with the same diagnosis. Previous studies have also highlighted that younger women often have less social support (Champion et al., 2014) and more responsibilities including young children and financial obligations. It is plausible that a combination of these clinical and social factors is driving the younger women’s more persistent (or repetitive) negative thoughts and COVID-EMV in our study as they likely have greater concerns about the impacts of COVID-19 on their long-term survivorship and the impact the outcomes could have on their family. Based on our findings we recommend that further qualitative research is conducted to explore the types of negative thoughts or concerns (i.e., recurrence) younger women are encountering as this will allow more informed support to be provided.

The findings from our study also indicate that self-reported physical illness comorbidity (as measured by CCI) is predictive of the severity of rumination and cognitive impairment experienced by women with breast cancer. This result is consistent with studies that have shown that poorer self-reported physical health has a significant association with rumination (Thomsen et al., 2004). These underlying physical conditions such as cardiovascular disease and diabetes are a crucial prognostic factor in early breast cancer (Land et al., 2012).

Finally, we found that after controlling for several clinical and sociodemographic variables such as education, grade, age at diagnosis, time since treatment finished, and comorbidity index, COVID-19 related emotional distress (COVID-EMV) was a notable predictor for more rumination, worry and cognitive impairment in women with a history of primary breast cancer. This result can be explained by the fact that women are experiencing an increasing number of negative feelings such as a sense of loss of control over their health due to the COVID-19 pandemic, with the added stress of disruptions to oncological services resulting in greater COVID-19 emotional vulnerability. Such negative feelings could be provoking cognitive impairment by creating an internal distraction and a greater level of worry and rumination which if not targeted early could then progress into clinical emotional disorders including anxiety and depression. Rumination is known to play a critical role in the development and maintenance of affective disorders (i.e., anxiety, depression or mixed anxiety and depression) (Nolen-Hoeksema, 2000). Emotional distress in breast cancer patients has been associated with unfavorable outcomes in treatment compliance (Greer et al., 2008), which might negatively impact on disease progression and mortality rate (Satin et al., 2009). With depression and anxiety both predicting cancer recurrence and cancer specific mortality in breast cancer patients it becomes imperative to address the cognitive and emotional vulnerability experienced by women as a result of COVID-19 to avoid any further detrimental effects on their long-term health.

The present study adds to the growing body of research indicating that groups with underlying health conditions are more likely to suffer adverse outcomes due to the COVID-19 outbreak (Jiang et al., 2020; Lai et al., 2020). Our results also extend those of Swainston et al. (2020), who also found that delays in treatment resulted in more COVID-EMV, and also worse COVID-EMV in primary breast cancer predicting more emotional (psychological) distress and cognitive impairments. Further studies which focus on the underlying factors in developing emotional and cognitive problems in the breast cancer population will need to be undertaken. Investment in online interventions such as cognitive training that have successfully reduced anxiety and rumination in breast cancer survivors (Swainston and Derakshan, 2018) is highly recommended. In addition, governmental efforts can target the development of applications designed to assist women with breast cancer to record and monitor their symptoms on a daily basis so that with online communication patients can relay information to healthcare professionals who in turn can identify those at higher risk of developing clinical levels of anxiety and depression as well as other physical conditions needing attention.

## Limitation

Our study is limited due to the cross-sectional design. Data were collected at a one-time point via a series of online questionnaires. Approximately 60% of our participants had higher education levels, which means that these findings may not be fully representative of Iran’s breast cancer population. Secondly, delayed treatment did not specify the amount of time lost due to delays mainly because participants were unaware of any rescheduling to their treatment at the time the study was conducted. Finally, our study focused on women with a primary diagnosis of breast cancer. Future studies need to investigate the collateral damage brought about by COVID-19 on the mental well-being of women with metastatic breast cancer who are at a greater risk of clinical vulnerability to depression.

## Conclusion

The most prominent finding to emerge from this study is that women with breast cancer are at risk of experiencing more rumination, worry and cognitive impairment due to COVID-19 related emotional distress. The critical role of worry and rumination in the maintenance of depression, anxiety and PTSD, as well as the role of depression and anxiety in cancer recurrence and mortality, indicate the possible risks facing women with breast cancer as a result of the COVID-19 outbreak. This study has highlighted the realities we continue to face from the psychological impact of the outbreak in vulnerable populations and individuals suffering from underlying health conditions. Further work can investigate the long-term impact of the COVID-19 pandemic on women living with breast cancer who will benefit from access to cognitive and emotional health interventions.

## Data Availability Statement

The raw data supporting the conclusions of this article will be made available by the authors, without undue reservation.

## Ethics Statement

The studies involving human participants were reviewed and approved by Jamshid Shanbehzadeh, Department of Electrical and Computer Engineering, Kharazmi University, I. R. Iran Azizollah Habibi Department of Chemistry, Kharazmi University, Tehran, Iran; Mehdi Abbasi Sarmadi, Faculty of Law and Political Sciences, Kharazmi University, Tehran, Iran; Ali Akbar Imani, Department of Islamic Studies, Faculty of Literature and Humanities, Tehran, Iran; Mahdi Akbarzadeh, The Cellular and Molecular Endocrine Research Center, Shahid Beheshti University of Medical Sciences, Tehran, Iran; Mehdi Tehrani-Doost, Department of Psychiatry, Tehran University of Medical Sciences, Tehran, Iran; Fatemeh Javaheri, Department of Sociology, Faculty of Literature and Humanities, Kharazmi University, Tehran, Iran; Jafar Hasani, Department of Clinical Psychology, Faculty of Psychology and Education, Kharazmi University, Tehran, Iran. Online informed consent to participate in this study was provided by the participant at the start of the study.

## Author Contributions

BC, EG, VM, and ND: research question and study design. AM: funding. MC and VM: preparation of data and data collection. MC, VM, and BC: drafting initial version of manuscript and drafting final version of manuscript. All authors contributed to the analysis plan, statistical analysis, and critical review of early and final versions of manuscript.

## Conflict of Interest

The authors declare that the research was conducted in the absence of any commercial or financial relationships that could be construed as a potential conflict of interest.
