# Supplementary material for: The Impact of COVID-19 Outbreak on Emotional and Cognitive Vulnerability in Iranian Women With Breast Cancer
Source: Front Psychol. 2021 May 31;12:663310. doi: 10.3389/fpsyg.2021.663310 (PMC8220448; doi:10.3389/fpsyg.2021.663310)
Supplement: Supplementary file 1 [file Table_1.DOCX]

**Supplementary Material**

Reliability analysis showed that COVID-EMV had excellent reliability, α = 0.90.

| Table 1. Inter-item correlation for COVID-EMV | | | | | |
| --- | --- | --- | --- | --- | --- |
|  | COVID-19 Anxious | COVID-19 Upset | COVID-19 Fearful | COVID-19 Control | COVID-19 Confident |
| COVID-19 Anxious | 1.00 |  |  |  |  |
| COVID-19 Upset | 0.72 | 1.00 |  |  |  |
| COVID-19 Fearful | 0.57 | 0.59 | 1.00 |  |  |
| COVID-19 Control | 0.61 | 0.66 | 0.78 | 1.00 |  |
| COVID-19 Confident | 0.50 | 0.66 | 0.63 | 0.66 | 1.00 |

| Table 2. Item-Total Statistics | | | | | |
| --- | --- | --- | --- | --- | --- |
|  | Scale Mean if Item Deleted | Scale Variance if Item Deleted | Corrected Item-Total Correlation | Squared Multiple Correlation | Cronbach's Alpha if Item Deleted |
| COVID-19 Anxious | 9.27 | 20.27 | 0.69 | 0.56 | 0.89 |
| COVID-19 Upset | 9.55 | 19.41 | 0.77 | 0.65 | 0.87 |
| COVID-19 Fearful | 9.86 | 18.96 | 0.76 | 0.64 | 0.87 |
| COVID-19 Control | 9.99 | 18.91 | 0.81 | 0.69 | 0.86 |
| COVID-19 Confident | 10.12 | 19.55 | 0.72 | 0.55 | 0.88 |

| Table 3. KMO and Bartlett's Test | | |
| --- | --- | --- |
| Kaiser-Meyer-Olkin | 0.83 |  |
|  |  |  |
|  | Bartlett's Test of Sphericity |  |
| Approx. Chi-Square | df | Sig. |
| 425.25 | 10.00 | 0.00 |

| Table 4. Factor Analysis for COVID-EMV | | |
| --- | --- | --- |
|  | Loading onto Factor One | Communalities |
| COVID-19 Anxious | 0.81 | 0.65 |
| COVID-19 Upset | 0.86 | 0.74 |
| COVID-19 Fearful | 0.85 | 0.72 |
| COVID-19 Control | 0.88 | 0.78 |
| COVID-19 Confident | 0.82 | 0.67 |
| Eigenvalue | 3.56 |  |
| % of Total Variance | 71.19 |  |
|  |  |  |

| Table 5. Correlations between COVID-EMV and cognitive and emotional health questionnaires | | | |
| --- | --- | --- | --- |
|  | Rumination  Response Scale | Penn State Worry | FACT-Cog |
| COVID-EMV | 0.60** | 0.48** | -0.40** |
| **Correlation is significant at the 0.01 level | | | |
